# Dataset on the relation of synthetic super para magnetic nanoparticles coated with various electric charges and fibrillation of albumin protein

**DOI:** 10.1016/j.dib.2018.10.170

**Published:** 2018-11-03

**Authors:** Negin Javdani, Sayyed Shahryar Rahpeyma, Younes Ghasemi, Jamshid Raheb

**Affiliations:** a National Institute of Genetic Engineering and Biotechnology, Tehran, Iran; b Pharmaceutical Sciences Research Center, Shiraz University of Medical Sciences, Shiraz, Iran

**Keywords:** Nanoparticle, Iron Oxide, Albumin, Fibrillation

## Abstract

The data provided in this article are related to the research article entitled “Effect of Super Magnetic Nanoparticles Coated with Various Electric Charges on α-Synuclein Protein Fibrillation Process” (Javdani et al.). This article describes how electrically different charged and concentrated iron oxide nanoparticles synthesized using reverse co-precipitation method affects the fibrillation of albumin protein.

**Specifications table**

**Table:** 

Subject area	*Biochemistry, Nanotechnology*
More specific subject area	*Relationship between nanoparticles and proteins*
Type of data	*Image (TEM), text file, graph, figure*
How data was acquired	*Super para magnetic nanoparticle were synthesized by revers co-precipitation method;*
	*The nanoparticles size and chemical properties were observed by TEM, VSM and XRD;*
	*The fibrillation of albumin was measured using THT*
Data format	*Analyzed*
Experimental factors	*The nanoparticles were synthesized using reverse co-precipitation method*
Experimental features	*The relationship between nanoparticles and fibrillation of albumin was investigated*
Data source location	*Iran, Tehran, National institute of Genetic Engineering and Biotechnology*
Data accessibility	*The data is provided within the article*
Related research article	*N. Javdani, S.S. Rahpeyma, Y. Ghasemi, J. Raheb. Effect of Super Magnetic Nanoparticles Coated with Various Electric Charges on α-Synuclein Protein Fibrillation Process. J Applied Nanoscience. (under review)*[1]

**Value of the data**

• The data indicates synthesize of nanoparticles by reverse co-precipitation method and it is possible to be used by other researchers.
• The fibrillation of albumin by the impact of different surficial charged nanoparticles was measured by using the THT and could be compared to other fibrillation sets of data.
• This set of data provides the opportunity for other researchers to have further extensive statistical studies in this regard.

## 1 Data

**Fig. 1:**
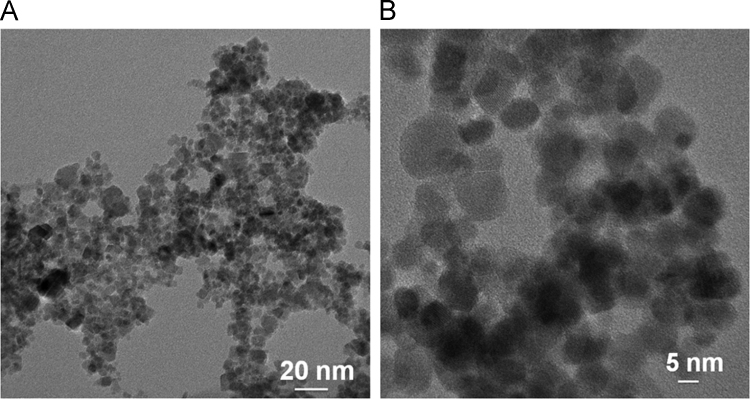
The size and chemical compositions of nanoparticles I (A) and II (B) were investigated using TEM.

**Fig. 2:**
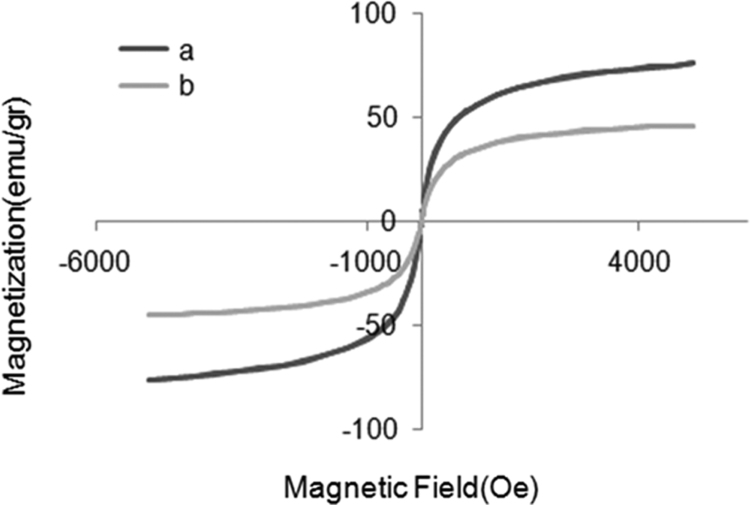
Magnetic saturation diagram of synthesized iron oxide nanoparticles type I (a) and II (b).

**Fig. 3:**
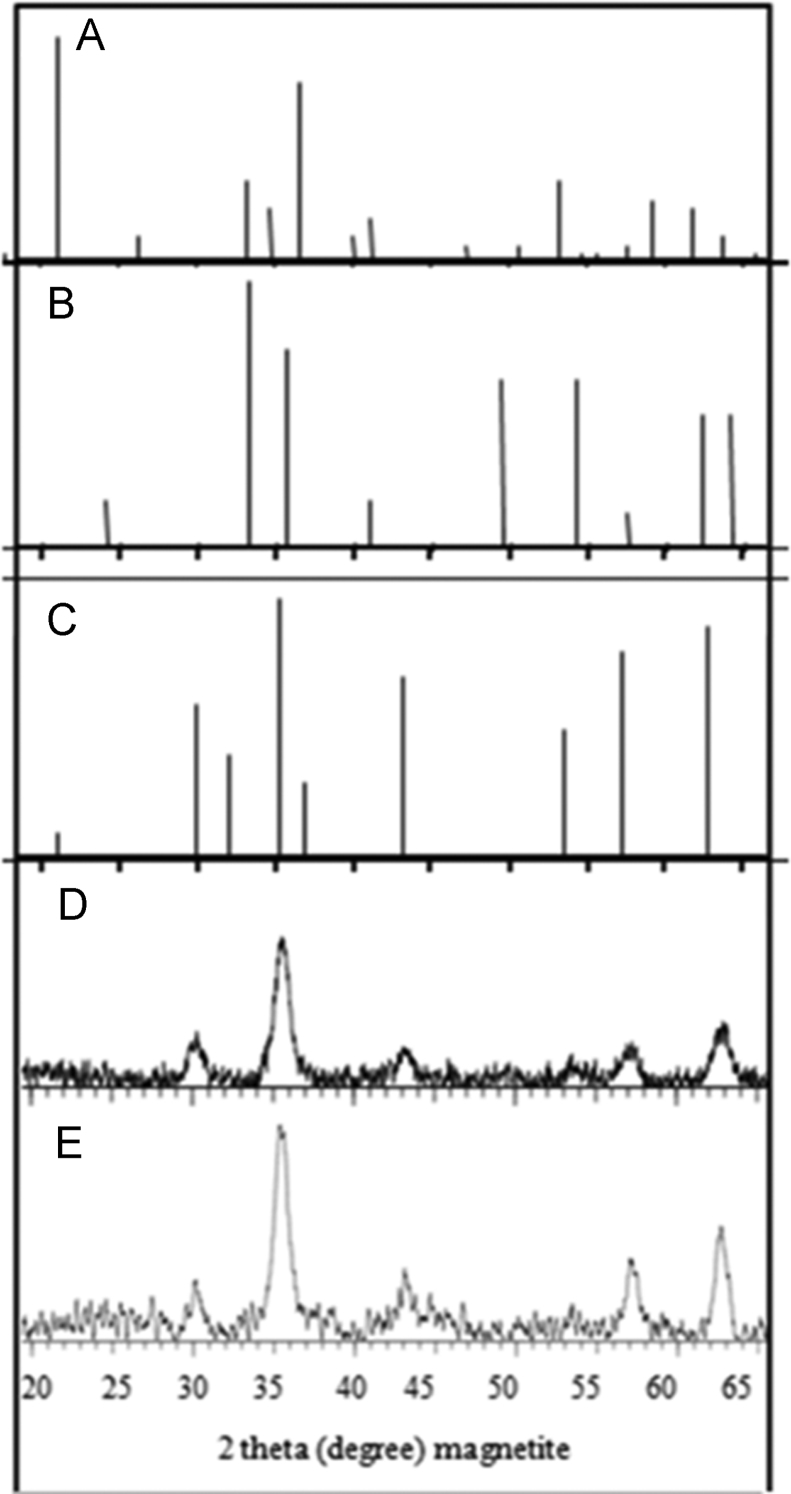
Goethite Standard Models (A) (α-FeO (OH)), Hematite (B) (α-Fe_2_O_3_), Magnetite (C) (Fe_3_O_4_) and XRD model of synthesized nanoparticle samples type I (D), II (E).

**Fig. 4:**
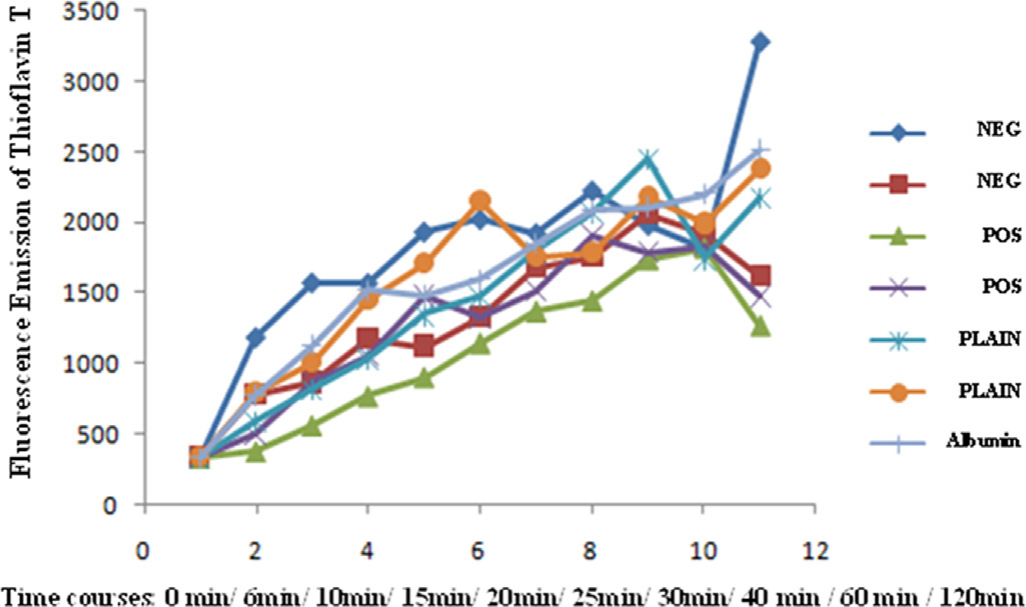
The relation between synthetic iron oxide nanoparticles with various surface charges and fibrillation of albumin investigated by THT.

This article contains the data on synthesize of iron oxide nanoparticles using reverse co-precipitation method. There is also information about the size and electrical features of the nanoparticles. As the main part, data of the influence of the synthesized nanoparticles with various surface electrical charges and concentrations on fibrillation process of albumin can be observed. Fig. 1 illustrates synthesized iron oxide nanoparticles type I and II TEM images. In Fig. 2 the magnetic saturation diagram of synthesized iron oxide nanoparticles type I and II is shown. Fig. 3 demonstrates diagrams of Goethite Standard Models (α-FeO (OH), hematite (α-Fe_2_O_3_), magnetite (Fe_3_O_4_) and XRD models of synthesized nanoparticle type I, II. Eventually Fig. 4 provides diagrams of albumin fibrillation using THT test.

## 2 Experimental Design, Materials and Methods

### 2.1 Super paramagnetic iron oxide nanoparticle synthesis

Iron oxide nanoparticles were synthesized using reverse co-precipitation method [2]. In this method, first, saline was prepared adding iron salts (III) and (II) with a molar ratio of 2:1 in 5 of deionized (DI) water and .17 of hydrochloric acid. Then, 50 ml ammonia-alkaline solution (pH = 10) was prepared. The saline was slowly added to alkaline solution and strongly blended with a mechanical stirrer at a rotation speed of 1,200 rpm while nitrogen was passing through it. Then, glycine was added as a surfactant in two ways. Iron oxide nanoparticles were synthesized using this method under two conditions. To synthesize iron oxide nanoparticles type I, glycine was added in two phases (during and after the synthesis). At first, 1.5 ml of glycine 0.27 g/ml and then after 20 min of stirring, 3 ml of glycine 0.4 g/ml were added. For synthesizing nanoparticles type II, only 3 ml of glycine 0.5 g/ml was added after 20 min of stirring. In general, the solution was placed in an ultrasonic bath for 20 min after 30 min of stirring. The prepared nanoparticles were dispersed in 40 ml of DI water after dissociation through magnet (three times) and washing in the water (deoxygenated). The final concentration of magnetite nanoparticles was 10 mg/l.

### 2.2 Magnetic power investigation of both synthesized nanoparticles

Magnetic powers of both synthesized nanoparticles (type I and II) were analyzed using VSM (Vibrating Sample Magnetometer) set. Their magnetic saturations were determined as 45/84 g/emu and 75/85 g/emu, respectively.

### 2.3 X-Ray diffraction (XRD) analysis

The XRD model obtained from dark powder of both synthesized nanoparticles shows that Fe_3_O_4_ crystals were mainly found in both samples. The size of both synthesized nanoparticles (types I and II) were estimated about 7.4 and 8.5 nm using Scherrer equation.

d=0.9λ/tcosθ

In this equation, d and λ denote the diameter and length of the nanoparticles, respectively. The X-ray wave input on the sample was equal to 1.54187 Å, t was equal to half-width of the highest peak and θ denoted for diffraction angle of X-ray.

### 2.4 The relation between iron oxide nanoparticles with various surface charges and fibrillation of albumin

The relation between iron oxide nanoparticles with different surface charges and fibrillation of albumin, was investigated by THT test. Different nanoparticles were first incubated with albumin protein and then heated at different time periods so that the protein was first solved in phosphate buffer (pH = 7.4) and then it was incubated at the presence of iron oxide nanoparticles with various surface charges at 65 °C. Finally, the amount of albumin-related fibril production was investigated using fluorimeter set [3].
