# Supplementary material for: Dataset on the relation of synthetic super para magnetic nanoparticles coated with various electric charges and fibrillation of albumin protein
Source: Data Brief. 2018 Nov 3;21:1853–7. doi: 10.1016/j.dib.2018.10.170 (PMC6260359; doi:10.1016/j.dib.2018.10.170)
Supplement: Supplementary file 1 — Supplementary material [file mmc1.docx]

Dear editor of DIB

There is no conflict of interests between authors.

Corresponding author: Jamshid Raheb
